# Spin Trapping Hydroxyl and Aryl Radicals of One-Electron Reduced Anticancer Benzotriazine 1,4-Dioxides

**DOI:** 10.3390/molecules27030812

**Published:** 2022-01-26

**Authors:** Wen Qi, Pooja Yadav, Cho R. Hong, Ralph J. Stevenson, Michael P. Hay, Robert F. Anderson

**Affiliations:** 1 School of Chemical Sciences, The University of Auckland, Private Bag 92019, Auckland 1142, New Zealand; wqi066@aucklanduni.ac.nz (W.Q.); pooja.yadav@auckland.ac.nz (P.Y.); 2 Auckland Cancer Society Research Centre, School of Medical Sciences, The University of Auckland, Private Bag 92019, Auckland 1142, New Zealand; c.hong@auckland.ac.nz (C.R.H.); rasikralph@gmail.com (R.J.S.); m.hay@auckland.ac.nz (M.P.H.); 3 Maurice Wilkins Centre for Molecular Biodiscovery, The University of Auckland, Private Bag 92019, Auckland 1142, New Zealand

**Keywords:** benzotriazine 1,4-dioxide, tirapazamine, hypoxia-activated prodrug, cytochrome P450 oxidoreductase, electron spin resonance, hydroxyl radical, aryl radical, cytotoxicity

## Abstract

Hypoxia in tumors results in resistance to both chemotherapy and radiotherapy treatments but affords an environment in which hypoxia-activated prodrugs (HAP) are activated upon bioreduction to release targeted cytotoxins. The benzotriazine 1,4-di-*N*-oxide (BTO) HAP, tirapazamine (TPZ, **1**), has undergone extensive clinical evaluation in combination with radiotherapy to assist in the killing of hypoxic tumor cells. Although compound **1** did not gain approval for clinical use, it has spurred on the development of other BTOs, such as the 3-alkyl analogue, SN30000, **2**. There is general agreement that the cytotoxin(s) from BTOs arise from the one-electron reduced form of the compounds. Identifying the cytotoxic radicals, and whether they play a role in the selective killing of hypoxic tumor cells, is important for continued development of the BTO class of anticancer prodrugs. In this study, nitrone spin-traps, combined with electron spin resonance, give evidence for the formation of aryl radicals from compounds **1**, **2** and 3-phenyl analogues, compounds **3** and **4**, which form carbon C-centered radicals. In addition, high concentrations of DEPMPO (5-(diethoxyphosphoryl)-5-methyl-1-pyrroline *N*-oxide) spin-trap the •OH radical. The combination of spin-traps with high concentrations of DMSO and methanol also give evidence for the involvement of strongly oxidizing radicals. The failure to spin-trap methyl radicals with PBN (*N*-*tert*-butylphenylnitrone) on the bioreduction of compound **2**, in the presence of DMSO, implies that free •OH radicals are not released from the protonated radical anions of compound **2**. The spin-trapping of •OH radicals by high concentrations of DEPMPO, and the radical species arising from DMSO and methanol give both direct and indirect evidence for the scavenging of •OH radicals that are involved in an intramolecular process. Hypoxia-selective cytotoxicity is not related to the formation of aryl radicals from the BTO compounds as they are associated with high aerobic cytotoxicity.

## 1. Introduction

**Figure 1:**
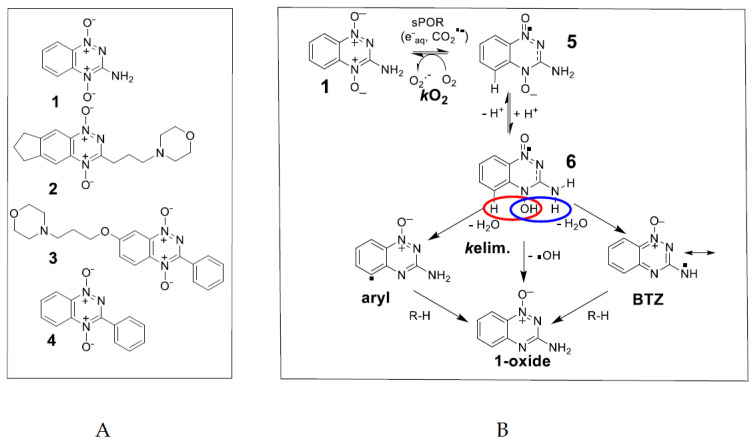
(**A**) Structures of compounds used in this study. (**B**) Scheme showing the activation of TPZ following oxygen-sensitive one-electron reduction to produce the radical anion intermediate and possible unimolecular routes of dehydration and homolytic fragmentation of the *N*-OH bond of its protonated form to produce aryl, BTZ and •OH radicals.

Tirapazamine, TPZ, (3-amino-1,2,4-benzotriazine-1,4 dioxide, **1**) (Figure 1A), is the most investigated example of the 1,2,4-benzotriazine 1,4-*N*-dioxide (BTO) class of bioreductive drugs, which are selectively cytotoxic against hypoxic tumor cells [1,2,3]. The mechanism by which BTO compounds are activated to cause oxidative damage to DNA continues to be the subject of considerable research. The stable 2-electron reduced (1-oxide) and 4-electron reduced (nor-oxide) metabolites of TPZ proved to be relatively non-toxic [4] and attention on the identity of the cytotoxin produced upon bioreduction by cellular reductases initially centered on the one-electron reduced form of TPZ, the radical anion **5** or its protonated form **6** (Figure 1B).

However, radical anions are reducing radicals and the TPZ radical anion is oxidized by molecular O_2_ to restore TPZ with concomitant production of superoxide, O_2_•^−^. Pulse radiolysis studies have shown that the back reaction is in competition with a first-order kinetic reaction to form an oxidizing radical species [5,6], which most likely results in increased cytotoxicity under hypoxia. The hypoxic cytotoxicity ratio (HCR) is defined as the ratio of drug concentrations giving equitoxic effects in an in vitro assay under normoxia and anoxia: HCR = IC_50oxic_/IC_50anoxic_. The HCR values for compound **1** typically fall in the range of 50–300 [7,8] depending on cell type, culture conditions and drug exposure, and compound **2** was shown to be consistently more selective across a panel of cell lines [9]. One early proposal for the oxidizing species was that the protonated radical anion of TPZ underwent homolytic fragmentation of the *N*^4^-OH bond to release the highly reactive •OH radical and the non-toxic 1-oxide metabolite [10,11,12,13] (Figure 1B). This mechanism is supported by the observation that oxidative damage to DNA occurs, although asymmetric purine over pyrimidine damage was observed [11,14].

An electron spin resonance (EPR) study, using the spin trap 5,5-dimethyl-1-pyrroline *N*-oxide (DMPO), supported the release of the •OH radical from the radical anion under aerobic conditions [15]. However, DMPO is subject to facile oxidation followed by nucleophilic addition of hydroxyl ions to give a false positive result for the •OH radical [16]. This was shown to be the case for TPZ in an experiment with H_2_^17^O where hydroxylation of the DMPO is sourced from the solvent and not from TPZ [16]. Dehydration of the protonated radical anion involving the N-H on the 3-NH_2_ substituent was proposed to form an oxidizing benzotriazinyl radical, BTZ [5] (Figure 1B), and a corresponding multi-*N*-centered radical for a soluble derivative of TPZ has been detected by EPR [17]. Substitution of the 3-NH_2_ substituent with a 3-alkyl solubilizing sidechain, as in SN30000, (3-(3-morpholinopropyl)-7,8-dihydro-6H-indeno[5,6-e][1,2,4]triazine 1,4-dioxide, **2**) [9,18] (Figure 1A) results in the formation of a benzodiazinyl radical, BDZ, upon similar dehydration with the radical character shared on the sidechain carbon. The radical one-electron reduction potential of BTZ, 1.31 V [5] and BDZ, 1.35 V [19] are strong enough to undergo one-electron oxidation reactions of purine bases of DNA, but not pyrimidine bases [20,21]. The fact that some damage to pyrimidine bases is observed points to another more highly reactive radical, or radicals, also being formed. Simulated EPR spectra of radicals formed upon anaerobic one-electron reduction of TPZ and SN30000, by NADPH cytochrome P450 oxidoreductase (sPOR) and spin-trapped by *N*-*tert*-butylphenylnitrone (PBN), give a wide H-atom hyperfine coupling constant (hfc), a*H* ≥ 4.0 G [19,22] as evidence for the formation of aryl-type radicals [23,24,25] (Figure 1B). DFT calculations for TPZ found water elimination involving the 3-NH_2_ substituent to be highly exothermic, with elimination involving the C5-H slightly endothermic and with •OH radical elimination from *N*^4^-OH being more endothermic. A similar pattern is calculated for analogues with 3-substituents of -H, -CH_3_ -phenyl and -OCH_3_. Substitution of C5-H with C5-OCH_3_ gave a*H* values comparable to a typical C-radical adduct of 3.70 G [22]. The proposed dehydration reaction involving the protonated *N*-oxide moiety and neighboring hydrogen on C5 to produce an aryl radical on C5 implies a π to σ-radical transition. Support for such a transition comes from a study with pyridinium compounds where the out-of-plane *N*-OR bond could allow mixing of π* and σ* orbitals and bond fragmentation [26]. However, there is only a small difference in overall energy between such a mechanism and the unimolecular *N*-OH bond homolysis, with subsequent H-atom abstract by the •OH radical in a caged-like aggregate. Thus it is difficult to distinguish between the two mechanisms from theoretical calculations alone [27].

In this study, we investigate the two possible mechanisms for aryl radical formation in compounds **1**, **2** and the water soluble (ca. 10 mM in culture medium) 3-phenyl substituted BTO compound, **3** (Figure 1A), by using EPR with high concentrations (≥100 mM) of the spin-traps PBN and DEPMPO (5-(diethoxyphosphoryl)-5-methyl-1-pyrroline N-oxide). DEPMPO, unlike PBN, forms a relatively stable •OH radical adduct. Compound **4** has poor aqueous solubility for EPR studies (ca. 1 mM), although an incomplete simulated spectrum with PBN as the spin trap indicated the presence of an aryl radical upon anaerobic reduction by sPOR [22]. The life-times of both DEPMPO and PBN adducts with aryl radicals enable their detection by EPR, and DEPMPO forms a persistent adduct with the •OH radical [28]. In contrast, the •OH-PBN radical adduct has a half-life < 1 min [29]. As both PBN and DEPMPO have high rate constants in scavenging •OH radicals (8.5 × 10^9^ M^−1^·s^−1^ [30] and 4.9 × 10^9^ M^−1^·s^−1^ [31], respectively), we reasoned that high concentrations of spin-traps may intercept a proportion of any •OH radical formation through unimolecular *N*-OH bond homolysis before intramolecular H-atom abstraction. High concentrations of the •OH radical scavengers DMSO (2 M) and methanol (1.5 M) may also act in a similar way. Physical chemistry properties of the radicals formed by compounds **3** and **4**, their reactivity, as well as in vitro cytotoxicity of the compounds arising from such radicals, are compared with data obtained for TPZ, **1** and SN30000, **2**. EPR spectra of spin-trapped radicals and radical property differences between all four compounds, enable insights into mechanistic drivers of cytotoxicity for the BTO class of anticancer compounds.

## 2. Results

### 2.1. Pulse and Steady-State Radiolysis Studies

The one-electron reduction potential, *E*^0’^, of compound **3** was measured at pH 7.0 using pulse radiolysis and found to be ca. 18 mV lower than that of compound **4**, Table 1, reflecting the electron-donating property of the 7-OR substituent. The 3-phenyl substituted compounds possess *E*^0’^ values significantly higher than compounds **1** (3-NH_2_) and compound **2** (3-alkyl, 6,7-cycloalkyl) which is reflected in lower radical anion reaction rate constants with O_2_ (see Supplementary Materials), Table 1. The radical anions of all four compounds underwent kinetic 1st-order reactions, *k*elim, in competition with bimolecular second-order reactions. Data obtained for compound **3** (Figure 1), together with previously determined values for compounds **1**, **2** and **4**, are presented in Table 1. Electron donation by the 7-substituent raises the *k*elim rate constant by an order of magnitude.

When compound **1** undergoes one-electron reduction in anoxia in the presence of H-donors (e.g., formate ions, deoxyribose), it is consumed by short chain reactions [6,22,32]. 

**Table 1:** Physical Chemistry Properties of BTO Compounds.

Compound	*E*^0’^(A/A^.−^)/mV	*k*elim/s^−1^	10^6^*k*O_2_/M^−1^ s^−1^	G-loss/μM.Gy^−1^
**1**	−456 ± 8 ^a,b^	83 ± 6 ^e^	6.20 ± 0.25 ^g^	1.55 ± 0.07
**2**	−401 ± 8 ^c^	125 ± 15 ^c^	3.33 ± 0.03 ^c^	0.95 ± 0.04
**3**	−387 ± 7	1530 ± 187	2.32 ± 0.17	1.96 ± 0.05
**4**	−369 ± 8 ^d^	150 ± 15 ^f^	2.10 ± 0.06	2.20 ± 0.06

^a^ [33], ^b^ [7], ^c^ [19], ^d^ [34], ^e^ [6], ^f^ [22], ^g^ [32].

**Figure 2:**
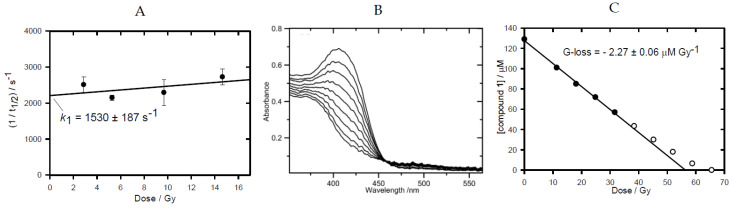
Radiation chemistry data. (**A**) Dependence of the elimination rate on the radiation dose, measured at 530 nm (k_1_ is the value of the intercept). The N_2_O-saturated solution (pH 7) contained compound **3** (100 µM) and sodium formate (0.1 M). (**B**) Stepwise changes in absorption spectrum with accumulated radiation dose in N_2_-saturated solution, containing compound **1** (129 μM), sodium formate (0.1 M) and phosphate buffer (2.5 mM) at pH 7. (**C**) Changes in absorption at 407 nm upon stepwise irradiation of compound **1**, as displayed in (**B**). The G-loss value is calculated from the linear regression fit to the initial points.

This is evidence for a strong oxidizing species being produced as well as another radical(s) which does not propagate the chain. The BTZ radical, the •OH radical and an aryl radical all must be proposed to be released from the protonated radical anion of compound **1** and are capable of H-atom abstractions. In this study, all four compounds were irradiated in N_2_-saturated solutions containing sodium formate (0.1 M) at pH 7 and changes in their UV-visible spectra with accumulated radiation doses followed. Isobestic points were maintained as starting spectra were converted into final product spectra, permitting the linear loss of compound with radiation dose to be determined (Figure 2B for compound **1**; see Supplementary Materials for compounds **2**-**4**) and hence G-loss values in μM.Gy^−1^ were obtained, Table 1. All compounds exhibited G-loss values greater than the G-value for the reductants (e^−^_aq_ + CO_2_•^−^ species) of 0.62 μM.Gy^−1^ [35], with the 3-phenyl substituted compounds having a chain length larger than that observed for compounds **1** and **2**.

### 2.2. EPR Experiments

The one-electron bioreduction of compounds **1**, **2**, and **3** by sPOR was carried out anaerobically at 37 °C in situ in the EPR spectrometer at pH 7.0 in the presence of high concentrations of the nitrone spin-traps. In general, spectra were accumulated over several scans to give satisfactory signal to noise ratios. Previous simulation of the spectrum obtained upon the reduction of compound **1** with added PBN (50 mM) gave evidence for the presence of two species; an aryl radical-PBN adduct and a C-centered radical-PBN adduct in the ratio 0.20:0.80 [22], Table 2. In addition, an experiment with added DEPMPO (25 mM) produced a spectrum which was well simulated by only a C-centered radical-DEPMPO adduct [17], Table 2. With such low concentrations of the spin-traps, the C-centered radicals may well arise from the released radicals reacting with the biochemical molecules present in solution rather than with the spin-traps. In an experiment with a higher concentration of DEPMPO (100 mM), a complex spectrum is obtained which is simulated as consisting of both a C-centered radical adduct and the •OH-DEPMPO adduct (see Supplementary Materials). 

**Figure 3:**
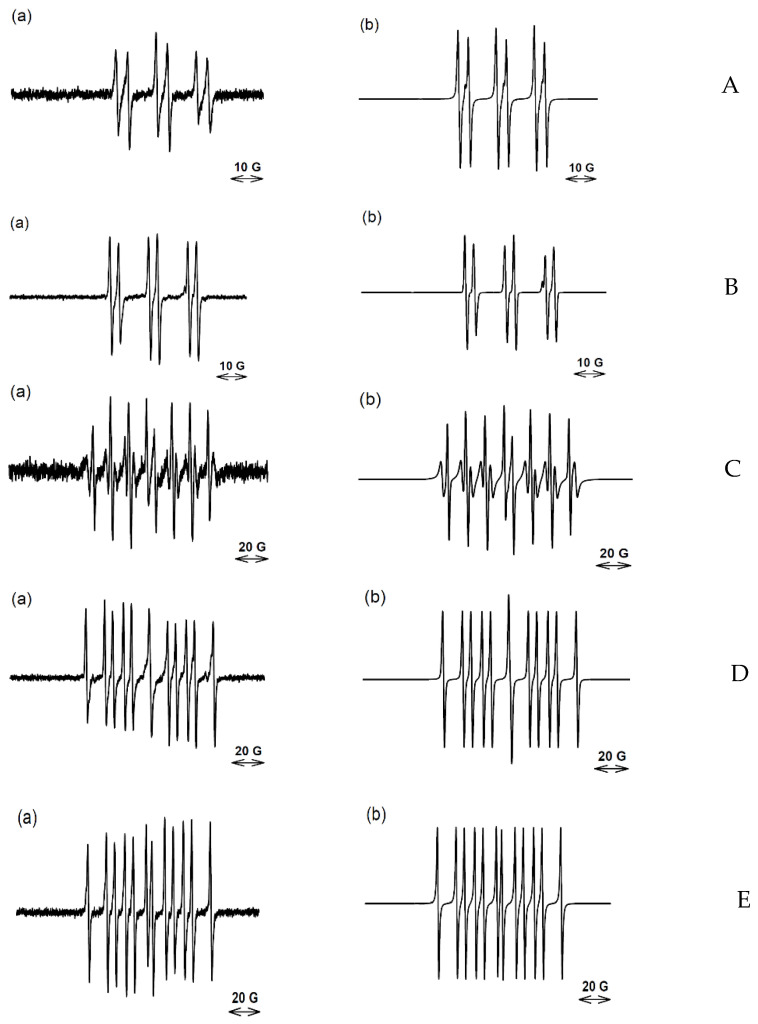
EPR spectra obtained for compound **2** and their simulation. (**A**) (**a**) EPR spectra (100 scans) obtained on reduction of compound **2** (17 mM) by POR (14 ng/mL) in anaerobic solutions at 37 °C containing phosphate buffer (50 mM, pH 7), DTPA (100 µM), SOD (300 units/mL), catalase (1500 units/mL), glucose-6-phosphate (10 mM), glucose-6-phosphate-dehydrogenase (13 units/mL), and NADPH (1 mM) in presence of PBN (250 mM); (**b**) simulated spectrum of PBN-aryl (0.91) and PBN-C-centered species (0.07), r = 0.94. (**B**) (**a**) EPR spectra (70 scans) obtained on reduction of compound **2** (16 mM) under the same conditions as for A with DMSO (2 M) added; (**b**) simulated spectrum of PBN-CH_2_(CH_3_)SO (0.74) and PBN-aryl (0.26), r = 0.99. (**C**) (**a**) EPR spectra (50 scans) obtained on reduction of **2** (18 mM) under the same conditions as for A with DEPMPO (250 mM); (**b**) simulated spectrum of DEPMPO-aryl (0.49), DEPMPO-OH (0.39) and DEPMPO-C-centered species (0.12), r = 0.97. (**D**) (**a**) EPR spectra (149 scans) obtained on reduction of compound **2** (16 mM) under the same conditions as for A with methanol (2.5 M); (**b**) simulated spectrum of DEPMPO-CH_2_OH, r = 0.98. (**E**) (**a**) EPR spectra (110 scan) of radicals obtained on reduction of compound **2** (15 mM) under the same conditions as for A with DMSO (2 M) added; (**b**) simulated spectrum of DEPMPO-carbon, r = 0.98.

Simulation of the spectrum previously obtained with bioreduced compound **2** with added PBN (50 mM) indicated the formation of both a C-centered radical-PBN adduct and an aryl radical-PBN adduct in the ratio of 43:57 [19] (Table 2). On increasing the concentration of PBN to 250 mM, simulation of the produced spectrum, after the same number of accumulated scans (100), indicated an increased percentage of the aryl radical and a decrease in the C-centered radical being spin-trapped (Figure 3). This indicates that the C-centered radical may arise from reaction of the aryl radical. An experiment with the same concentration of PBN (100 mM), but with DMSO (2 M) added, gave a spectrum simulated by an aryl radical-PBN adduct and a species with hfc of a*N* 16.50 G, a*H* 3.62 G, after 70 accumulated scans (Figure 3, Table 2). The •OH radical is known to be scavenged by high concentrations of DMSO through adding to the sulfur moiety and releasing the methyl radical [36], which is spin-trapped to form the CH_3_-PBN adduct. However, the above hfc do not match the methyl radical spin-trapped by PBN (a*N* 15.07 G, a*H* 3.42 G) [37], but do match the hfc of the •CH_2_(CH_3_)SO radical adduct of PBN [38]. At 137 accumulated scans, only this adduct was seen. These results could mean that a strong oxidant other than the •OH radical, such as an aryl radical, is formed and abstracts an H-atom from the methyl groups of DMSO. Aryl radicals have high C-H bond dissociation energies, BDE (e.g., phenyl radical, 118 kcal mol^−1^) compared to aliphatic C-H BDE (96–100 kcal mol^−1^) [39]. However, the possibility exists that •OH radicals, if formed, are completely scavenged by the high concentration of DMSO (2 M) before they can abstract the H-atom at C5 of the BTO. The released methyl radicals from DMSO undergo the known H-atom abstraction from the methyl groups of DMSO [40] to form the •CH_2_(CH_3_)SO species trapped by PBN. Similar considerations can be applied to the result of an experiment with DEPMPO. Previously, a C-centered radical derived from compound **2** was spin-trapped using 25 mM of DEPMPO, [19]. An initial concurrent, short-lived transient 8-line •OH-DEPMPO spectrum was also reported but dismissed as an artefact when a subsequent experiment with added DMSO gave a similar result. In this study, an experiment with compound **2**, in the presence of a high concentration of DEPMPO (250 mM), also produced a concurrent but persistent 8-line •OH-DEPMPO spectrum as part of an overall spectrum. After 50 scans, this spectrum is simulated well by a mixture consisting of mainly an aryl radical, the •OH radical and a minor amount of a C-centered radical (Figure 3C). In later scans, the percentage of spin-trapped aryl radicals decreases while the percentage of C-centered radical increases and the •OH-DEPMPO adduct persists (data not shown). An experiment with added methanol (2.5 M) resulted in the •CH_2_OH radical being exclusively spin-trapped by DEPMPO, (Figure 3D). Both the •OH radical and aryl radical can abstract an H-atom from the methyl group of methanol [41,42] to form the •CH_2_OH radical which is spin-trapped by DEPMPO with characteristic hfc [28]. An experiment with DEPMPO (250 mM) in the presence of DMSO (2 M) resulted in a C-centered radical being spin-trapped (Figure 3D). The hfc values of a*N* 15.25 G, a*H* 22.0 G, a*P* 48.14 G match those of a general C-centered radical [43], rather than those of the •CH_3_-DEPMPO spectrum, a*N* 15.2 G, a*H* 22.3 G, a*P* 47.7 G [44]. Small differences in the hfc of C-centered radicals trapped by DEPMPO, Table 3, may arise from the different adjacent structures to the radicals.

**Figure 4:**
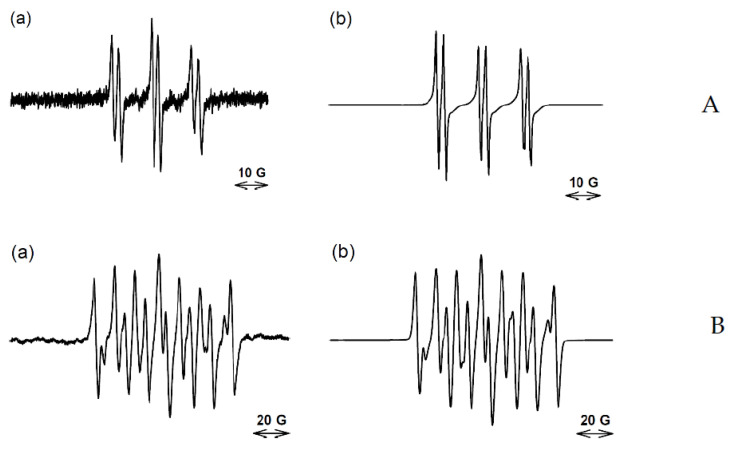
EPR spectra obtained for compound **3** and their simulation. (**A**) (**a**) EPR spectra (150 scans) obtained on reduction of compound **3** (10 mM) under the same conditions as Figure 3A with POBN (100 mM); (**b**) simulated spectrum of POBN-aryl (0.75) and POBN-C-centered species (0.25), r = 0.96. (**B**) (**a**) EPR spectra (125 scans) obtained on reduction of compound **3** (14 mM) under the same conditions as Figure 3A with DEPMPO (100 mM); (**b**) simulated spectrum of DEPMPO-C-centered species (0.65) and DEPMPO-OH (0.35), r = 0.99.

Simulation of a poor signal to noise spectrum obtained with reduced compound **3** and 20 mM PBN indicated the formation of both an aryl and carbon-centered radicals (spectrum not shown). An improved spectrum was obtained with 100 mM of the PBN analogue, α-(4-pyridyl-1-oxide)-*N*-tert-butylnitrone), 4-POBN (Figure 4A), where simulation supports a high proportion of spin-trapped aryl radical with an indicative a*N* 15.41 G a*H* 3.20 G [45,46], which is different from C-centered radicals [47,48], Table 2. With 100 mM DEPMPO in solution, only C-centered and •OH radicals were spin-trapped (Figure 4B, Table 2).

### 2.3. Cytotoxicity Data

Complete sets of in vitro cytotoxicity data under aerobic and anoxic conditions for compounds **1**, **2** and **3** were obtained for a head-to-head comparison along with published data (under the same conditions) for compound **4**, Table 3. Compound **4** showed high cytotoxic potency under aerobic conditions with little to no selectivity for hypoxic cells in vitro leading to HCR in HT29 and SiHa cancer cells of ca. 1.0 [34], and no selectivity in HCT116 cells. Our in vitro cytotoxicity data for compounds **1** and **2** is in agreement with the literature for HT29 and SiHa cell lines, e.g., [9]. The 3-phenyl group on the BTO nucleus (compounds **3** and **4**) completely ablates the hypoxic selectivity seen for compounds **1** (3-amino) and **2** (3-(4-morpholinyl)propyl) through increased oxic cytotoxic potency. 

## 3. Discussion

Bioreduction of the BTO compounds produced a range of radicals which were spin-trapped by DEPMPO, dependent on the concentration of the spin-trap. At a concentration of 25 mM only general C-centered radicals were trapped (compounds **1** and **2**), at 100 mM both C-centered and •OH radicals were spin-trapped (compounds **1** and **3**) while at 250 mM, aryl radicals were spin-trapped, in addition to C-centered radicals and the •OH radical (compound **2**). These results imply that at least some of the C-centered radicals arise from reactions of more reactive radicals and high concentrations of the spin trap are able to intercept these reactive radicals. This conclusion is supported by the results obtained using PBN where, at 50 mM PBN, mainly a C-centered radical was spin-trapped as well as an aryl radical upon bioreduction of compound **2**, whereas with 250 mM, >90% aryl radical was spin-trapped. It must be borne in mind that the percentages of spin-trapped species in Table 2 relate only to the radicals spin-trapped by DEPMPO and PBN and do not take into account other radicals which may be formed, such as non-trapped *N*-centered radicals, and in the case of PBN, •OH radicals, because the •OH-PBN adduct is extremely short-lived. Furthermore, the proportion of aryl radical to •OH radical spin-trapped by 250 mM DEPMPO initially increased with time (scan number), but then steadily decreased in time with a concomitant rise of a C-centered radical. This is most likely related to the aryl radicals reacting with biomolecules in the reduction milieu in competition to being spin-trapped and the aryl-DEPMPO adduct being kinetically less stable than the C-centered DEPMPO adduct.

**Figure 5:**
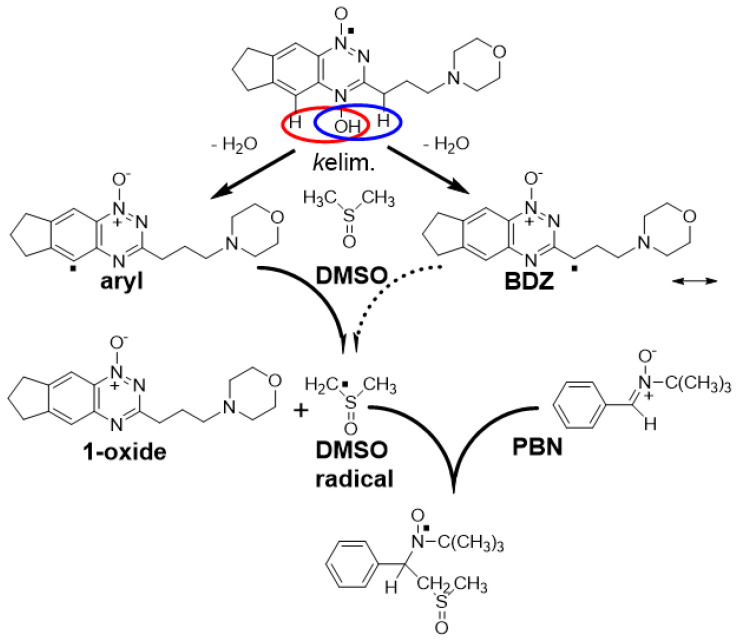
Scheme showing the formation of the aryl radical and BTZ radicals from the protonated radical anion of compound **2**, their possible reactions with DMSO to form a C-centered radical on DMSO and its spin-trapping by PBN to form the •CH_2_(CH_3_)SO-PBN adduct.

Only DEPMPO formed a detectable adduct with the •OH radical in this study, near the limit of its solubility at 250 mM. This being the case, it was not possible to test if the aryl radical arises from an intramolecular abstraction reaction by the •OH radical released from the protonated radical anions of BTO compounds by varying the concentration of DEPMPO. Both DMSO and methanol are highly water-soluble and are efficient •OH radical scavengers producing unique trappable radicals, and these scavengers were employed in an attempt to answer this question. In the case of DMSO, methyl radicals (•CH_3_) are spin-trapped as a surrogate for the •OH radical. The fact that the •CH_3_-PBN adduct was not seen, but rather the •CH_2_(CH_3_)SO-PBN adduct was observed on bioreduction of compound **2** (Figure 5), is evidence against the free formation of the •OH radical from the protonated radical anions of the BTO compounds. However, the concentration of DMSO in the experiment was 2 M and such a conclusion relies on the assumption that the pseudo first-order rate constant (s^−1^), *k*abstraction(•CH_3_ + DMSO) × 2 M ˂˂ *k*addition(•CH_3_ + PBN) × 0.1 M. It is a reasonable assumption to make as the rate constants for methyl radical addition to unsaturated bonds are over an order of magnitude greater than H-atom abstraction in aqueous solution to form methane [49].

Interpretation of the result of the experiment with DEPMPO (250 mM), in combination with DEPMPO (2 M), is not definitive because the experimental hfc cannot be clearly assigned to a general C-centered radical-DEPMPO and the CH_3_-DEPMPO adducts. In addition, the hfc for the •CH_2_(CH_3_)SO species trapped by DEPMPO has not been reported, which would have aided interpretation. Both the •OH radical and aryl radicals abstract an H-atom from methanol to form •CH_2_OH which is spin-trapped by DEPMPO with definitive hfc. The absence of the any detectable •CH_3_-PBN species produced following the bioreduction of compound **2** in the presence of DMSO, implies that free •OH radicals are not released from its protonated radical anion. This conclusion leads to the suggestion that the detection of the •OH radical by spin-traps (in the present study, DEPMPO) arises from an internal scavenging mechanism. The absence of the aryl-DEPMPO species in the presence of •OH radical scavengers does not imply that the aryl radical is not formed by an intramolecular H-abstraction reaction by the •OH radical because aryl radicals can undergo H-atom abstraction reactions with the scavengers as does the •OH radical.

The fact that one-electron reduction of all four compounds lead to the maintenance of UV-visible spectral isobestic points throughout the irradiations indicates a clean transition to stable products. The UV-visible spectra of the products are well matched to the 1-oxide derivatives of the compounds. G-loss values for all four compounds exceed the G-value of reductants upon their radiolytic reduction in the presence of formate ions (Table 1) meaning that short chain reactions are occurring. This can be explained by one (or more) of the radicals formed upon the breakdown of the radical anions being able to abstract an H-atom from formate ions forming CO_2_•^−^ which propagates the chain. This can be expressed as: G_R_ + xG_R_ + x^2^G_R_ + … + x^n^G_R_ = -S where -S is the G-loss of a compound, G_R_ is the G-value of the sum of the reductants (e^−^_aq_ + CO_2_•^−^), x is the fraction of reduced compound that goes into the cycle of the chain reaction. The series can be represented by ∑^∞^_n_ = 0.xn = 1/(1 − x) where x = 1 + G_R_/S. Results obtained for solving this equation for x and y (fraction of reductants that do not propagate the chain) are given in Table 4. Compound 2 containing the 3-alkyl sidechain exhibited the shortest radical chain length. One hypothesis is that aryl radicals can abstract an H-atom from formate ions to form the CO_2_•^−^ radical, which propagates the chain. As x is the minor fraction, it can be inferred that the protonated radical anion of compound **2** preferentially undergoes dehydration to initially form a C-centered radical at C1 on the sidechain which is ring stabilized as a BDZ radical. For compound **1**, aryl radical formation slightly dominates over BTZ radical formation. The protonated radical anions of compounds **3** and **4** could form two different aryl-type radicals, suggested to be either on C5 of the benzotriazine ring, or on the 3-phenyl substituent. As the radiolytic loss of these compounds is due to a short chain reaction, the two types of aryl radicals must have different reactivity with respect to H-atom abstraction from formate ions. Taking into account the results with compounds **1** and **2**, it is likely that the aryl radical on the benzo ring is responsible for propagating the chain.

The 3-phenyl substituted BTO compounds, **3** and **4**, do not display hypoxic selectivity because of increased aerobic cytotoxicity. This could arise because of the formation of highly reactive aryl-type radicals formed via dehydration of their protonated radical anions, or by intramolecular H-atom abstraction by the •OH radical. Aryl radicals react with O_2_ to form peroxyl radicals [50] which may lead to the formation of alkoxyl radicals (*E*^0’^ 1.77 V [51]) and be more cytotoxic than the primary radicals produced by bioreduction of BTO compounds. Qualitative conclusions can be drawn on the nature of radicals formed upon the reduction of BTO compounds that underlie HCR values. In contrast to compounds **3** and **4**, which have HCR values ~1, compounds **1** (3-NH_2_) and 2 (3-R) exhibit large HCRs. This may be related to the formation of *N*-centered radicals (BTZ for compound **1**) and C/N-centered radicals (BDZ for 2). All four compounds produced aryl radicals, which may be more potent than either the BTZ or BDZ radicals, however, they are not associated with large HCR values.

## 4. Materials and Methods

### 4.1. Materials 

**Figure 6:**
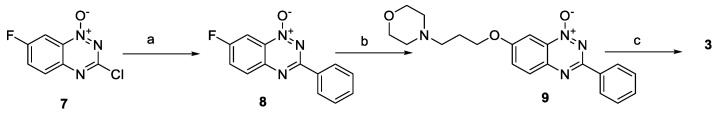
Synthesis of dioxide compound **3**. Reagents: (a) Phenylboronic acid, PdCl_2_.dppf.DCM, K_2_HPO_4_, DMF, water; (b) 3-(4-morpholinyl)propanol, NaH, THF; (c) H_2_O_2_, (CF_3_CO)_2_O, CF_3_CO_2_H, CHCl_3_.

TPZ [52], **1**, SN30000 [18], 2, and compound **4** [34], were synthesized as previously described. 7-(3-morpholinopropoxy)-3-phenylbenzo[*e*][1,2,4]triazine 1,4-dioxide (compound **3**) was prepared from 3-chloro-7-fluorobenzo[*e*][1,2,4]triazine 1-oxide (compound **7**) (Figure 6).

7-Fluoro-3-phenylbenzo[*e*][1,2,4]triazine 1-oxide (compound **8**). PdCl_2_.dppf.DCM (61 mg, 75 µmol) was added to a stirred, degassed solution of 3-chloro-7-fluorobenzo[*e*][1,2,4]triazine 1-oxide (compound **7**) [53] (300 mg, 1.51 mmol), phenylboronic acid (240 mg, 1.96 mmol) and K_2_HPO_4_ (2 M, 7 mL) in DMF (25 mL) and the mixture was stirred under N_2_ at 80 °C for 2 h. The mixture was partitioned between EtOAc (100 mL) and water (100 mL), the organic fraction was washed with water (2 × 50 mL), dried, and the solvent was evaporated. The residue was purified by chromatography, eluting with a gradient (0–10%) of EtOAc/pet. ether, to give 1-oxide compound **8** (207 mg, 57%) as a white solid: mp (EtOAc/pet. ether) 176–179 °C; ^1^H NMR (CDCl_3_) δ 8.51 (dd, *J* = 7.8, 2.0 Hz, 2 H, H-2′, H-6′), 8.11–8.17 (m, 2 H, H-5, H-8), 7.73 (ddd, *J* = 8.3, 7.9, 2.8 Hz, 1 H, H-6), 7.52–7.60 (m, 3 H, H-3′, H-4′, H-5′); ^13^C NMR (CDCl_3_) δ 163.8, 161.2, 160.6 (d, *J* = 3 Hz), 145.2, 134.0, 132.2, 132.1 (d, *J* = 9 Hz), 129.1 (2), 128.6 (2), 126.2 (d, *J* = 16 Hz), 105.5 (d, *J* = 17 Hz). Anal. calcd for C_13_H_8_FN_3_O: C, 64.73; H, 3.34; N, 17.42. Found: C, 64.69; H, 3.30; N, 17.55%.

7-(3-Morpholinopropoxy)-3-phenylbenzo[*e*][1,2,4]triazine 1-oxide (compound **9**). A solution of 3-(4-morpholinyl)propanol [54] (210 mg, 1.43 mmol) in dry THF (2 mL) was added dropwise to a stirred suspension of NaH (60% dispersion in oil, 76 mg, 1.91 mmol) in dry THF (15 mL) at 20 °C and the mixture was stirred for 30 min. A solution of fluoride compound **8** (230 mg, 0.95 mmol) in THF (2 mL) was added and the resulting solution was stirred at 70 °C for 4 h. The reaction was cooled to 0 °C, carefully quenched with water (5 mL) and the mixture partitioned between EtOAc (50 mL) and water (50 mL). The organic fraction was washed with water (3 × 25 mL), washed with brine (25 mL). The combined organic fraction was dried, and the solvent evaporated. The residue was precipitated from a mixture of MeOH/DCM and then recrystallised from EtOAc/hexane to give 1-oxide compound **9** (310 mg, 89%) as a pale yellow solid: mp 145–147 °C; ^1^H NMR (CDCl_3_) δ 8.47–8.52 (m, 2 H, H-2′, H-6′), 7.98 (d, *J* = 8.2 Hz, 1 H, H-5), 7.78 (d, *J* = 2.8 Hz, 1 H, H-8), 7.57 (dd, *J* = 8.2, 2.8 Hz, 1 H, H-6), 7.50–7.55 (m, 3 H, H-3′, H-4′, H-5′), 4.23 (t, *J* = 6.4 Hz, 2 H, CH_2_O), 3.75 (t, *J* = 4.7 Hz, 4 H, 2 × CH_2_O), 2.55 (t, *J* = 7.1 Hz, 2 H, CH_2_N), 2.48–2.52 (m, 4 H, 2 × CH_2_N), 2.06 (m, 2 H, CH_2_); ^13^C NMR (CDCl_3_) δ 160.2, 159.1, 144.2, 134.5, 134.3, 131.7, 130.7, 129.5, 129.0 (2), 128.3 (2), 98.6, 67.8, 67.2 (2), 55.4, 54.0 (2), 26.2. Anal. calcd for C_20_H_22_N_4_O_3_·0.15hexane: C, 66.17; H, 6.40; N, 14.77. Found: C, 65.78; H, 6.45; N, 14.44%.

7-(3-Morpholinopropoxy)-3-phenylbenzo[*e*][1,2,4]triazine 1,4-dioxide (compound **3**). Hydrogen peroxide (70%; 1.42 mL, ca. 12.6 mmol) was added dropwise to a stirred solution of TFAA (2.6 mL, 251 mmol) in DCM (10 mL) at 5 °C (CAUTION: exotherm). The solution was stirred at 20 °C for 10 min, then cooled to 5 °C, added to a solution of 1-oxide **9** (460 mg, 1.26 mmol) and TFA (0.72 mL, 6.28 mmol) in DCM (10 mL) at 5 °C. The solution was stirred at 20 °C for 72 h, cooled to 5 °C, and water (5 mL) added. cNH_3_ solution was added dropwise to the vigorously stirred mixture until the mixture was basic and then stirred for 30 min. The mixture was extracted with CHCl_3_ (4 × 10 mL) and the combined organic fraction was dried and the solvent evaporated. The residue was purified by column chromatography, eluting with a gradient (0–5%) MeOH/EtOAc to give (i) unreacted starting material **9** (122 mg, 27%); and (ii) 1,4-dioxide compound **3** (60 mg, 13%) as a yellow solid: mp 148–151 °C; ^1^H NMR (CDCl_3_) δ 8.52–8.57 (m, 3 H, H-5, H-2′, H-6′), 7.69 (d, *J* = 2.6 Hz, 1 H, H-8), 7.61 (dd, *J* = 9.4, 2.7 Hz, 1 H, H-6), 7.52–7.58 (m, 3 H, H-3′, H-4′, H-5′), 4.27 (t, *J* = 6.4 Hz, 2 H, CH_2_O), 3.74 (t, *J* = 4.6 Hz, 4 H, 2 × CH_2_O), 2.56 (t, *J* = 7.0 Hz, 2 H, CH_2_N), 2.49 (t, *J* = 4.3 Hz, 4 H, 2 × CH_2_N), 2.06 (pent, *J* = 6.7 Hz, 2 H, CH_2_); ^13^C NMR (CDCl_3_) δ 162.1, 148.4, 136.4, 135.7, 132.0, 130.1 (2), 129.0, 128.5 (2), 128.4, 121.9, 100.3, 68.1, 67.1 (2), 55.2, 53.9 (2), 26.1. HRMS calcd for C_20_H_23_N_4_O_4_ (MH^+^) 383.1714, found 383.1710 (0.9 ppm). HPLC purity 96.3%.

*N*-*tert*-butyl-α-phenylnitrone (PBN) was obtained from Fluka, DEPMPO (5-(diethoxyphosphoryl)-5-methyl-1-pyrroline *N*-oxide) was obtained from Cayman Chemicals, and *β*-nicotinamide adenine dinucleotide phosphate (NADPH) from Applichem. Other chemicals were obtained from Sigma-Aldrich. Highly purified NADPH:cytochrome P450 oxidoreductase (sPOR) was prepared as previously described [19].

### 4.2. Radiation Chemistry

Time-resolved optical absorption and kinetic studies were carried out using the University of Auckland’s 4 MeV linear accelerator (Auckland, New Zealand) which delivers 200 ns electron pulses of typically 3 Gy dose. The optical detection system and method of dosimetry have been described [55]. The radiolysis of water produces three well-characterized reactive radical species used to initiate radical reactions, as well as molecular products (G values in μM produced per absorbed dose of 1 Gy (J Kg^−1^) are given in parenthesis).

H_2_O ^^^^→ e_aq_^−^ (0.28) + •OH (0.28) + •H (0.055) + H_2_ (0.04) + H_2_O_2_ (0.07) + H_3_O^+^ (0.28)

One electron reduction of compounds **3** and **4** was carried out by (i) the e_aq_^−^ in the presence of 2-methylpropan-2-ol (0.2 M) to scavenge the oxidizing radicals, and (ii) electron transfer from the CO_2_•^−^ species (*E*(CO_2_/CO_2_•^−^) = −1.90 V [56]) in N_2_O-saturated solutions (to quantitatively convert the e^−^_aq_ to •OH) containing 0.1 M sodium formate, which in turn is oxidized by •OH and •H atoms to give the reducing CO_2_•^−^ radical.

e_aq_^−^ + A → A•^−^ (AH•)

•OH (•H) + (CH_3_)_3_COH → •CH_2_(CH_3_)_2_COH + H_2_O (H_2_)

N_2_O + e_aq_^−^ → •OH + OH- + N_2_

•OH (•H) + HCOO- → H_2_O (H_2_) + CO_2_•^−^

CO_2_•^−^ + A → A•^−^ + CO_2_

The one-electron reduction potentials of the compound 3, *E*(A/A•^−^), vs. NHE, was determined at pH 7.0 (2.5 mM phosphate buffer) under conditions (i) by establishing redox equilibria in anoxia within 50 μs between three mixtures of the one-electron reduced compounds and the reference compound methylviologen (*E*(MV^2+/^MV•^+^) = −447 ± 7 mV) and calculating Δ*E* values from the equilibrium constants, *K*e, using the Nernst equation, as described in the literature [57]. The kinetic 1st-order decay of the radical anions under conditions (ii), *k*elim., was separated out from their second-order bimolecular decay by determining the first half-life of the decay in the absorption of the radical anion, t0.5 and plotting 1/t0.5 against radiation dose (radical concentration), where *k*elim. is determined from the intercept of the plot [5]. The rate constants for the back oxidation of the radical anions of the compounds **3** and **4** by O_2_, *k*O_2_, were determined from the slopes of the plots of the pseudo first-order rate constants for the loss in absorbance of the radical anions against a range of O_2_ concentrations in solution, as previously described [32]. Steady-state radiolysis was used to determine the G-loss value of compound **3** upon reduction by the CO_2_•^−^ radical using a ^137^Cs γ-source delivering a dose rate of ca. 2 Gy min^−1^. The loss of compound **3** was followed by monitoring the sequential changes in the UV-vis spectrum with radiation dose as previously described [6] in N_2_O-saturated solutions of sodium formate (0.1 M) at pH 7 (5 mM phosphate). G-loss values in μM Gy^−1^ (μmol J^−1^) were calculated using a G value for CO_2_•^−^ radical production under these conditions of 0.68 μM Gy^−1^ [35].

### 4.3. Methodology for EPR Experiments

EPR experiments were carried out within a TE011 cavity on a JEOL (JES-FA-200) EPR spectrometer (JEOL Ltd., Tokyo, Japan), equipped with a variable temperature controller (ES-DVT4), and operated at 9.1 GHz and a 100 KHz field modulation. All solutions were prepared in Milli-Q water pre-treated with Chelex-100 resin to remove trace amounts of polyvalent metal ions and diethylenetriaminepentaacetic acid (DETAPAC) was added to the solutions to suppress any Fenton-type chemistry. Aqueous solutions of compounds **1**, **2**, and **3** (in phosphate buffer, 50 mM at pH 7.4), NADPH, and the spin-traps (PBN or DEPMPO), were pre-degassed separately with N_2_ gas. Human NADPH:cytochrome P450 oxidoreductase (POR) superoxide dismutase (SOD) from bovine erythrocytes, glucose-6-phosphate (G-6-P), glucose-6-phosphate dehydrogenase (G-6-P-D) and the spin-traps were added into the sample vial under N_2_ atmosphere. All additions were carried out on ice and the enzyme reaction started by the addition of NADPH. An EPR flat quartz cell, designed for the variable temperature Dewar, was used for all measurements. Solutions for EPR measurements were transferred under N_2_ from the sample vial to the EPR cell, which had been flushed previously with N_2_, and inserted into the EPR cavity. Extreme care was taken to keep the system de-aerated at all times. The temperature of the cavity was raised to 37 °C to activate the enzyme incubation. EPR spectra, recorded at a power of 20 mW, were averaged (ca. 100 scans) to improve the signal-to-noise ratio over a scan range of 200 Gauss (G) at a modulation width of 0.5 G, scan time of 2 min, and time constant of 0.03 s. Computer simulation of spectra were carried out using the WINSIM EPR program (Bethesda, Rockville, MD, USA) available in the public domain of the NIEHS EPR database. The correlation coefficient, r, for all the spectral simulations was obtained.

### 4.4. In Vitro Cytotoxicity

Human HT29 and HCT116 colorectal carcinoma cells and SiHa cervical carcinoma cells were authenticated in-house by short tandem-repeat profiling. Cells were grown in α-MEM medium with 5% fetal bovine serum, without antibiotics, and were confirmed mycoplasma-free using a PCR-ELISA method (Roche Diagnostics). Log phase cells were seeded into 96 well plates at 1100 cells/well for HT29 and HCT116 and at 1500 cells/well for SiHa, allowed to attach for 2 h, then test compounds were added by dilution from DMSO stocks to give a top concentration of <1% DMSO before serial 3-fold dilution in the plates. After 4 h, cultures were washed 3 times with fresh medium and grown for a further 5 days before staining with sulforhodamine B to determine IC_50_ values (concentration to inhibit 50% growth) as previously described [58]. For anoxic exposure to compounds, cells were pelleted by centrifugation, transferred to a Pd-catalyst anaerobic chamber (Bactron-II, Shell Lab), resuspended in anoxic medium and exposed to drugs as above, but using medium and plates that had been equilibrated in the chamber for at least 3 days. After drug washout, cells were grown and stained as for the aerobic IC_50_ assays. The absorbance of aerobic and hypoxic controls (8 wells/plate) had a mean of 0.19 (CV 14.7%) and 0.13 (CV 18.7%) for aerobic and hypoxic exposures, respectively, across all assays. 

## 5. Conclusions

High concentrations of DEPMPO spin-trap the •OH radical together with aryl radicals arising from one-electron reduced BTO compounds. Indirect evidence from results with the spin trap PBN in the presence of DMSO, support a mechanism where the •OH radical is not released free in solution but undergoes an intramolecular reaction which can be intercepted by scavengers. 3-Phenyl-BTO compounds **3** and **4** produce aryl radicals on bioreduction and do not display hypoxic selectivity. Hence it can be ruled out that aryl radicals give rise to hypoxia-selective cytotoxicity observed for other BTO compounds as aryl radical formation is associated with high aerobic cytotoxicity.

## Figures and Tables

**Table 2:** Hyperfine Coupling Constants for Spin-Trapped Radical Species.

Compd	Spin Trap	Scans	Radical Adduct	a*N* /G	a*H^β^*/G	a*P*/G	%	r
**1**	PBN 50 mM ^a^	10	-aryl	16.50	4.18		20	0.96
(4 mM)			-carbon	16.18	3.45		80	
	DEPMPO 25 mM ^b^	20	-carbon	14.70	21.40	47.40	100	0.92
	DEPMPO 105 mM	20	-carbon -OH	15.01 14.15	22.30 13,22	47.90 47.56	89.8 10.2	0.88
**2**	PBN 50 mM ^c^	100	-aryl	16.0	4.20		43	0.96
(12 mM)			-carbon	16.2	3.44		57	
	PBN 250 mM	100	-aryl	16.03	4.27		91	0.94
(16 mM)			-carbon	16.13	3.44		7	
	PBN 100 mM + DMSO 2 M	70	-CH_2_(CH_3_)SO -aryl	16.50 15.88	3.62 4.18		74 26	0.99
	PBN 100 mM + DMSO 2 M	137	-CH_2_(CH_3_)SO	16.50	3.62		100	0.99
	DEPMPO 25 mM	30	-carbon ^c^	15.2	22.1	48.8	100	0.92
	DEPMPO 250 mM	50	-aryl	14.64	22.47	45.75	62	0.96
			-carbon	15.10	21.78	47.50	10	
			-OH	14.12	13.25	47.53	28	
	DEPMPO 250 mM + methanol 2.5 M	149	-CH_2_OH	14.80	21.19	49.59	100	0.98
	DEPMPO 25 mM + DMSO 2 M	110	-carbon	15.25	22.00	48.14	100	0.98
**3**	PBN 100 mM	150	-aryl	15.07	4.02		79	0.81
(10 mM)			-carbon	16.05	3.57		21	
	4-POBN 100 mM	150	-aryl	15.41	3.20		75	0.96
			-carbon	15.65	2.69		25	
	DEPMPO 100 mM	125	-carbon	14.62	22.02	47.18	65	0.99
			-OH	14.08	13.58	47.62	35	

^a^ [22], ^b^ [17], ^c^ [19].

**Table 3:** In vitro Inhibitory Cytotoxicity (IC50 /mM) and HCR of BTO Compounds.

#	HT29 Oxic	HT29 Anoxic	HT29 HCR	SiHa Oxic	SiHa Anoxic	SiHa HCR	HCT116 Oxic	HCT116 Anoxic	HCT116 HCR
**1**	411 ± 18	5.69 ± 0.58	72.2	140 ± 8.0	2.80 ± 0.27	50	91.7 ± 4.4	1.69 ± 0.66	54
**2**	375 ± 89	2.88 ± 0.22	130	245 ± 27	1.42 ± 0.32	173	126 ± 11	2.05 ± 0.66	61
**3**	4.07 ± 1.04	2.83 ± 0.50	1.44	1.93 ± 0.22	1.09 ± 0.27	1.77	1.36 ± 0.23	1.64 ± 0.06	0.83
**4**	6.9 ^a^	4.4 ^a^	1.56	2.1 ^a^	2.2 ^a^	1.0	0.84 ± 0.12	1.67 ± 0.52	0.5

**#** Compound, ^a^ [34].

**Table 4:** Loss of Compounds Upon Radiolysis.

Compound	G-loss μM.Gy^−1^	x	y	Fraction x of G-Value
**1**	1.55 ± 0.07	0.60	0.40	0.93
**2**	0.95 ± 0.04	0.35	0.65	0.33
**3**	2.20 ± 0.06	0.68	0.32	1.33
**4**	1.96 ± 0.05	0.72	0.28	1.58

## Data Availability

Not applicable.

## Supplementary Materials

The following are available online. One-electron reduction potential determination of compound **3**, Figure S1: Rate constant for O_2_ reaction with the radical anion of compound **3**, Figure S2: EPR spectrum of one-electron reduced compound **1** in the presence of DEPMPO, Figures S3–S5: Spectra and G-loss values of compounds **2**–**4** upon steady-stare radiolysis, Figures S6–S12: EPR control experiments: NMR data for compounds, **8**, **9** and **3.**

## Sample Availability

Samples of the compounds are not available from the authors.
