# Supplementary material for: Spin Trapping Hydroxyl and Aryl Radicals of One-Electron Reduced Anticancer Benzotriazine 1,4-Dioxides
Source: Molecules. 2022 Jan 26;27(3):812. doi: 10.3390/molecules27030812 (PMC8840461; doi:10.3390/molecules27030812)
Supplement: Supplementary file 1 [file molecules-27-00812-s001.zip › molecules-1509558-SI.pdf]

Supplementary Materials:

Spin Trapping Hydroxyl and Aryl Radicals of One-Electron Reduced Anticancer Benzotriazine 1,4-Dioxides

Wen Qi, Pooja Yadav, Cho R. Hong, Ralph J. Stevenson, Michael P. Hay and Robert F. Anderson

Contents: Pages

A. One-electron reduction potential of compound **3**. 1
B. Reaction rate constant of one-electron reduced compound **3** with O_2. 2
C. EPR spectrum of DEPMPO-spin-trapped radicals of one-electron reduced compound **1.** 2
D. G-loss determinations for compounds **2** – **4** upon stepwise steady-state radiolysis. 3-4
E. Control EPR experiments. 4-6
F. NMR data for new compounds, **8**, **9** and **3**. 7-12

A. One-electron reduction potential of compound **3**.

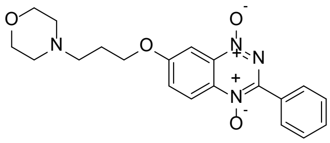

Redox equilibrium between methylviogen (MV^{2+}) as the redox indicator ($E(MV^{2+}/MV^{+\cdot})$ = -447 ±7 mV) and compound **3** (A) were established in N_2-saturated solutions containing 2-methylpropan-2-ol (0.2 M), MV^{2+} (1-3 mM), compound **3** (0.1-0.5 mM) at pH 7 (phosphate, 2.5 mM). Following pulse radiolysis (2.5 Gy in 200 ns), MV^{2+} and A were reduced by e_{aq}- and the equilibrium (1) observed at 600 nm to be established within 50 μs.

$$MV^{+\cdot} + A \rightleftharpoons MV^{2+} + A^{\cdot-} \quad (1)$$

From the experimental equilibrium constant, K =14.24 ± 1.45, ΔE = 68 ±3 mV is calculated using the Nernst equation, and making the calaculated correction for ionic strength of 8 mV, yielded the value E(A/A.-) = -387 ±7 mV.

B. Reaction rate constant of one-electron reduced compound **3** with O_2.

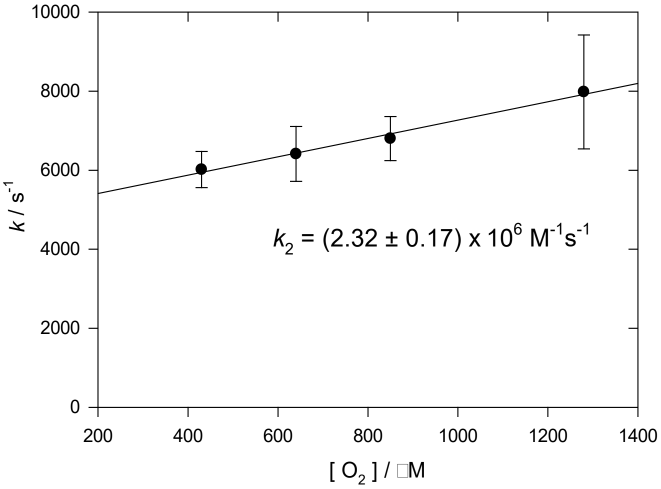

Figure S1. Dependence of the 1st-order rate constants for the decay of the radical anion of compound **3** on the concentration of O_2 following pulse radiolysis (ca. 10 Gy in 200 ns) of solutions saturated with O_2/N_2O mixtures, containing compound **3** (170 µM), sodium formate (0.1 M) and sodium phosphate (2.5 mM) at pH 4.5 (●), observed at 530 nm (The 2nd-order rate constant, k_2, is derived from the slope of the graph).

C. EPR spectrum of PBN spin-trapped radicals of one-electron reduced compound **2.**

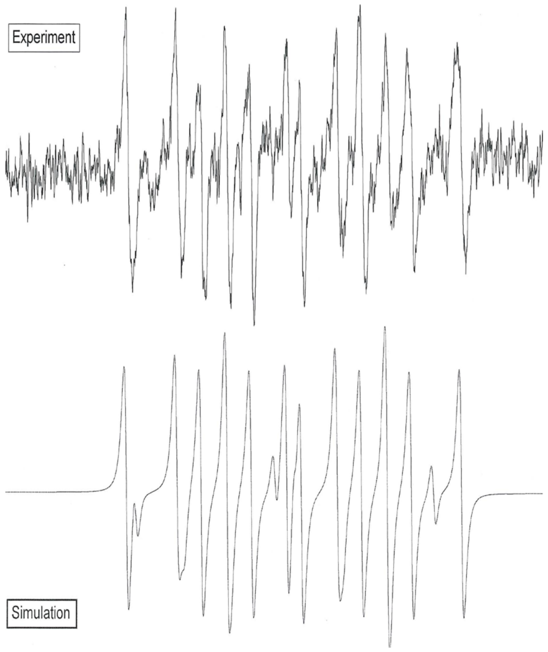

Figure S2. (a) EPR spectra (20 scans) obtained on reduction of compound **1** (17 mM) by POR (14 ng/mL) in anaerobic solutions at 37 °C containing phosphate buffer (50 mM, pH 7), DTPA (100 µM), SOD (300 units/mL), catalase (1500 units/mL), glucose-6-phosphate (10 mM), glucose-6-phosphate-

dehydrogenase (13 units/mL), and NADPH (1 mM) in presence of DEPMPO (105 mM); (b) simulated spectrum of DEPMPO-C centred species (0.90) and DEPMPO-OH centred species (0.10), r = 0.88.

D. G-loss determinations for compounds **2** – **4** upon stepwise steady-state radiolysis.

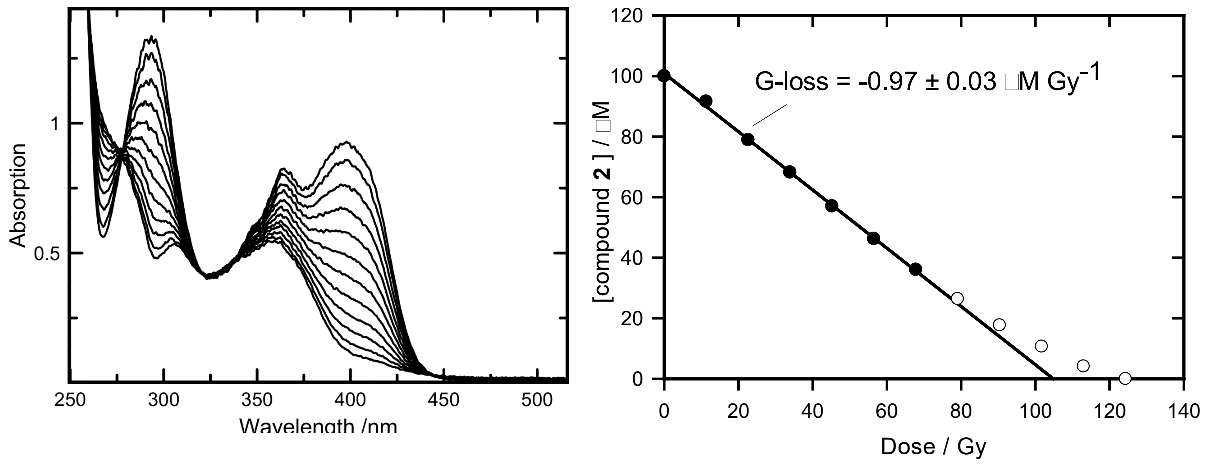

Figure S3. LHS Stepwise changes in absorption spectrum of compound **2** (SN30000) (100 μM) with accumulated radiation dose in N_2-saturated solution containing sodium formate (0.1 M) and phosphate buffer (2.5 mM at pH 7. RHS Change in the concentration of **2** (measured at 300 nm) with accumulated radiation dose. The G-loss value is calculated from the linear regression fit to the initial points of the graph.

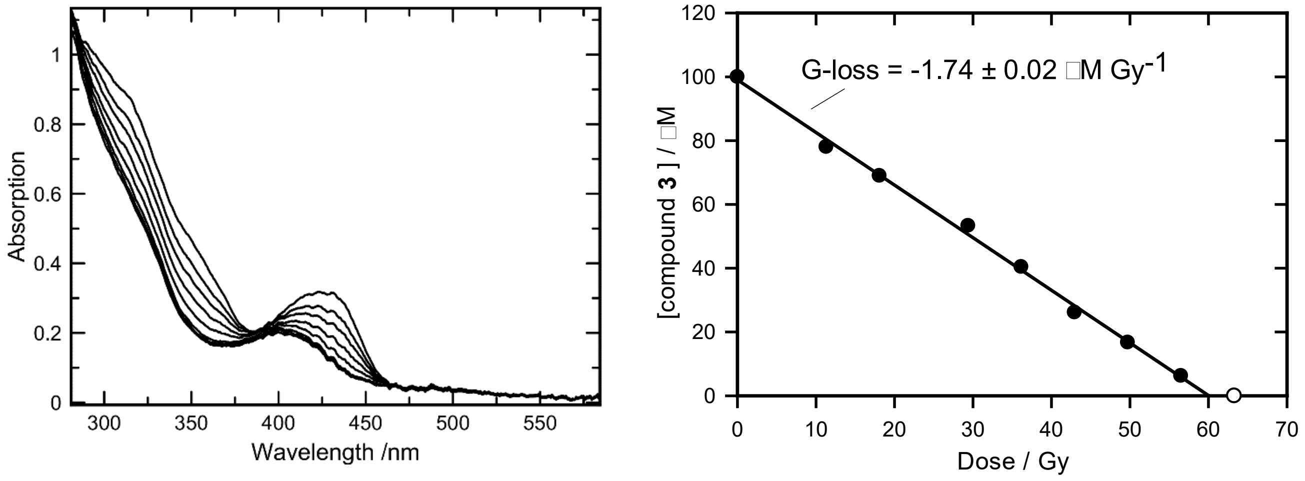

Figure S4. LHS Stepwise changes in absorption spectrum of compound **3** (100 μM) with accumulated radiation dose in N_2-saturated solution containing sodium formate (0.1 M) and phosphate buffer (2.5 mM at pH 7. RHS Change in the concentration of compound **3** (measured at 350 nm) with accumulated radiation dose. The G-loss value is calculated from the linear regression fit to the graph.

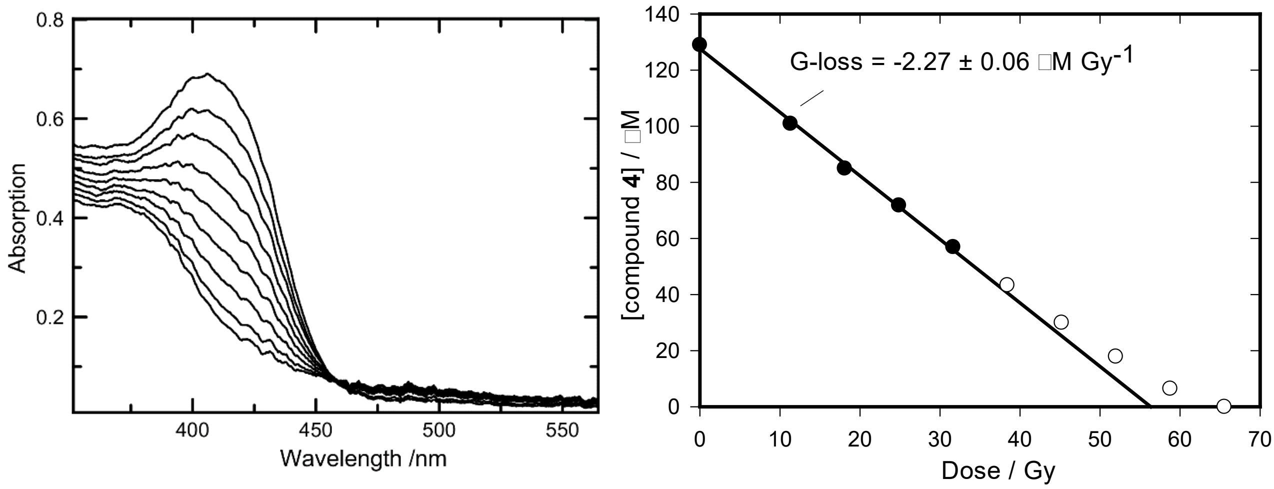

Figure S5. LHS Stepwise changes in absorption spectrum of compound **4** (129 μM) with accumulated radiation dose in N_2-saturated solution containing sodium formate (0.1 M) and phosphate buffer (2.5 mM at pH 7. RHS Change in the concentration of compound **4** (measured at 407 nm) with accumulated radiation dose. The G-loss value is calculated from the linear regression fit to the initial points of the graph.

E. Control EPR experiments.

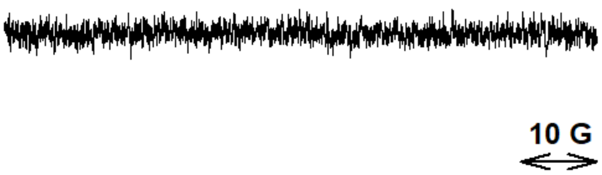

Figure S6. EPR spectrum obtained in anaerobic solution at 310 K containing phosphate buffer (50 mM, pH 7), POR (14 ng/mL), DTPA (100 μM), SOD (300 units/mL), catalase (1500 units/mL), glucose-6-phosphate (10 mM), and glucose-6-phosphate-dehydrogenase (13 units/mL).

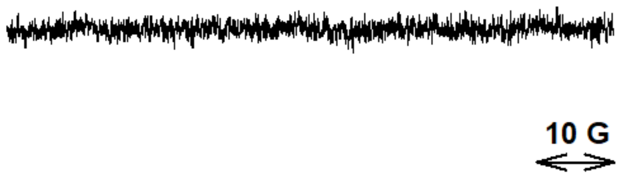

Figure S7. EPR spectrum obtained in anaerobic solution at 310 K containing phosphate buffer (50 mM, pH 7), POR (14 ng/mL), DTPA (100 µM), SOD (300 units/mL), catalase (1500 units/mL), glucose-6-phosphate (10 mM), glucose-6-phosphate-dehydrogenase (13 units/mL), and NADPH (1 mM).

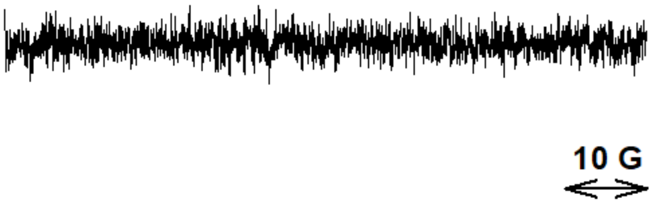

Figure S8. EPR spectrum obtained in anaerobic solution at 310 K containing phosphate buffer (55 mM, pH 7) and PBN (50 mM).

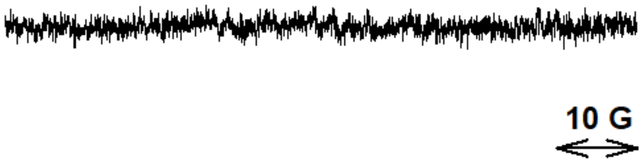

Figure S9. EPR spectrum obtained in anaerobic solution at 310 K containing phosphate buffer (50 mM, pH 7), POR (14 ng/mL), DTPA (100 µM), SOD (300 units/mL), catalase (1500 units/mL), glucose-6-phosphate (10 mM), glucose-6-phosphate-dehydrogenase (13 units/mL), and NADPH (1 mM) in presence of PBN (50 mM).

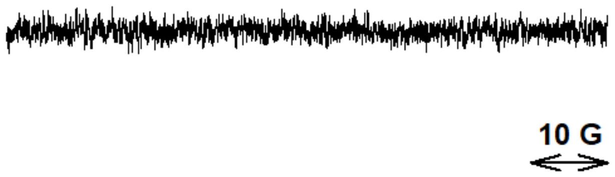

Figure S10. EPR spectrum obtained in anaerobic solution at 310 K containing phosphate buffer (50 mM, pH 7) and POBN (33 mM).

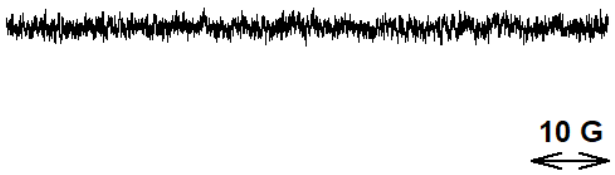

Figure S11. EPR spectrum obtained in anaerobic solution at 310 K containing phosphate buffer (50 mM, pH 7), POR (14 ng/mL), DTPA (100 µM), SOD (300 units/mL), catalase (1500 units/mL), glucose-6-phosphate (10 mM), glucose-6-phosphate-dehydrogenase (13 units/mL), and NADPH (1 mM) in presence of POBN (30 mM)

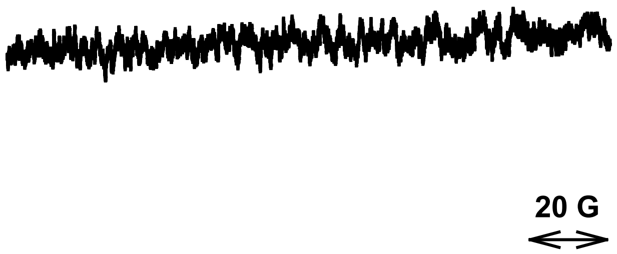

Figure S12. EPR spectrum obtained in anaerobic solution at 310 K containing phosphate buffer (50 mM, pH 7), POR (14 ng/mL), DTPA (100 µM), SOD (300 units/mL), catalase (1500 units/mL), glucose-6-phosphate (10 mM), glucose-6-phosphate-dehydrogenase (13 units/mL), and NADPH (1 mM) in presence of DEPMPO (25 mM).

Compound 8

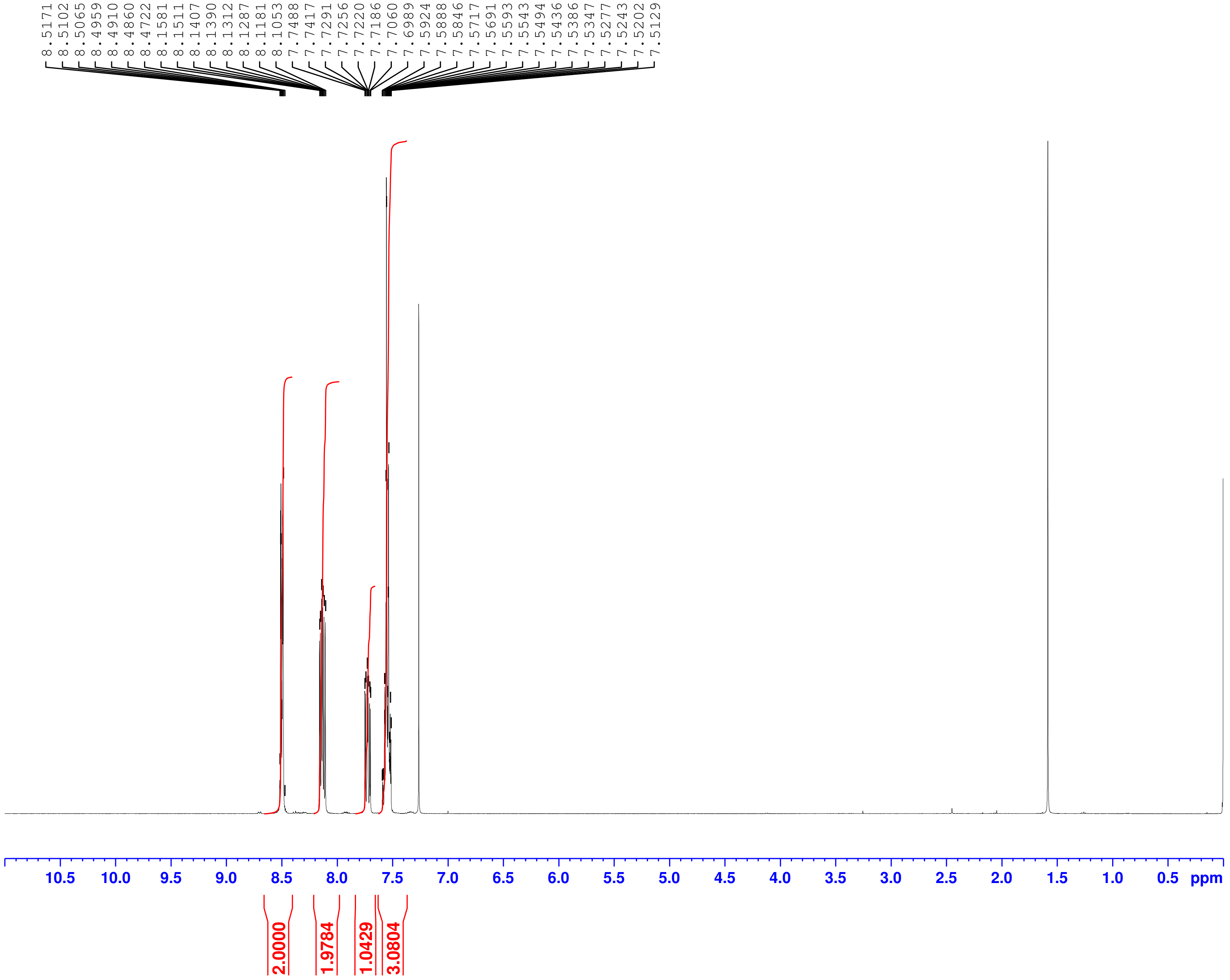

Current Data Parameters

NAME	Jan14-2022
EXPNO	18
PROCNO	1

F2 - Acquisition Parameters

Date_	20220116
Time	2.28 h
INSTRUM	spect
PROBHD	Z108618_0860 (
PULPROG	zg30
TD	65536
SOLVENT	CDCl3
NS	64
DS	2
SWH	8012.820 Hz
FIDRES	0.244532 Hz
AQ	4.0894465 sec
RG	198.55
DW	62.400 usec
DE	6.50 usec
TE	298.0 K
D1	1.00000000 sec
TD0	1
SFO1	400.1324708 MHz
NUC1	1H
P0	4.53 usec
P1	13.60 usec
PLW1	13.19999981 W

F2 - Processing parameters

SI	65536
SF	400.1300085 MHz
WDW	EM
SSB	0
LB	0.30 Hz
GB	0
PC	1.00

Compound 8

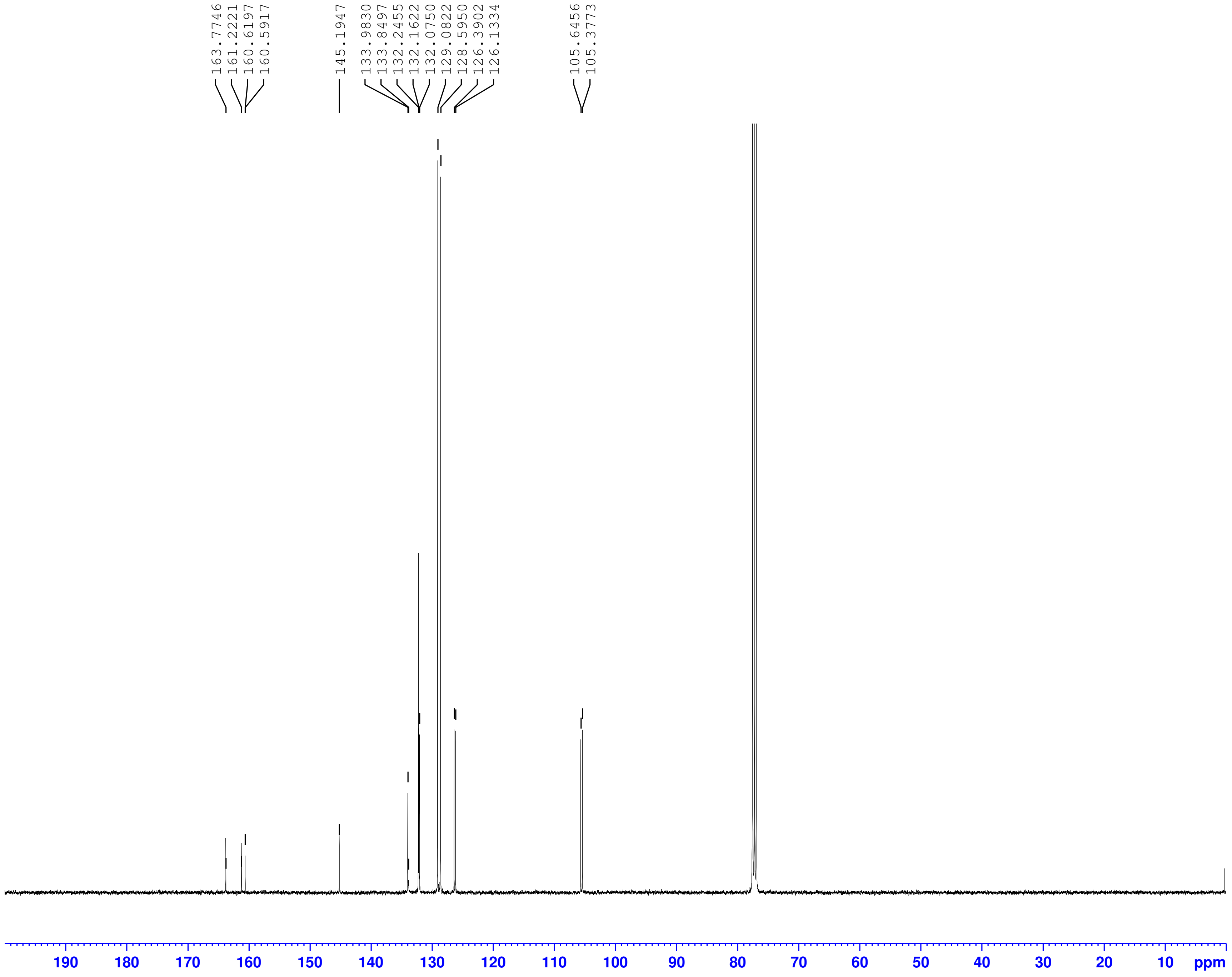


```
Current Data Parameters
NAME          Jan14-2022
EXPNO                 19
PROCNO                 1

F2 - Acquisition Parameters
Date_          20220116
Time              11.40 h
INSTRUM           spect
PROBHD   Z108618_0860 (
PULPROG          zgpg50
TD                65536
SOLVENT           CDCl3
NS                16000
DS                    4
SWH           24038.461 Hz
FIDRES         0.733596 Hz
AQ            1.3631488 sec
RG               198.55
DW               20.800 usec
DE                 6.50 usec
TE                298.0 K
D1           0.63999999 sec
D11          0.03000000 sec
TD0                   1
SFO1        100.6228298 MHz
NUC1                13C
P1                10.00 usec
PLW1        48.17399979 W
SFO2        400.1316005 MHz
NUC2                 1H
CPDPRG[2        waltz16
PCPD2             90.00 usec
PLW2        13.19999981 W
PLW12        0.30142000 W
PLW13        0.15161000 W

F2 - Processing parameters
SI                32768
SF          100.6127485 MHz
WDW                  EM
SSB                   0
LB                 1.00 Hz
GB                    0
PC                 1.40
```

Compound 9

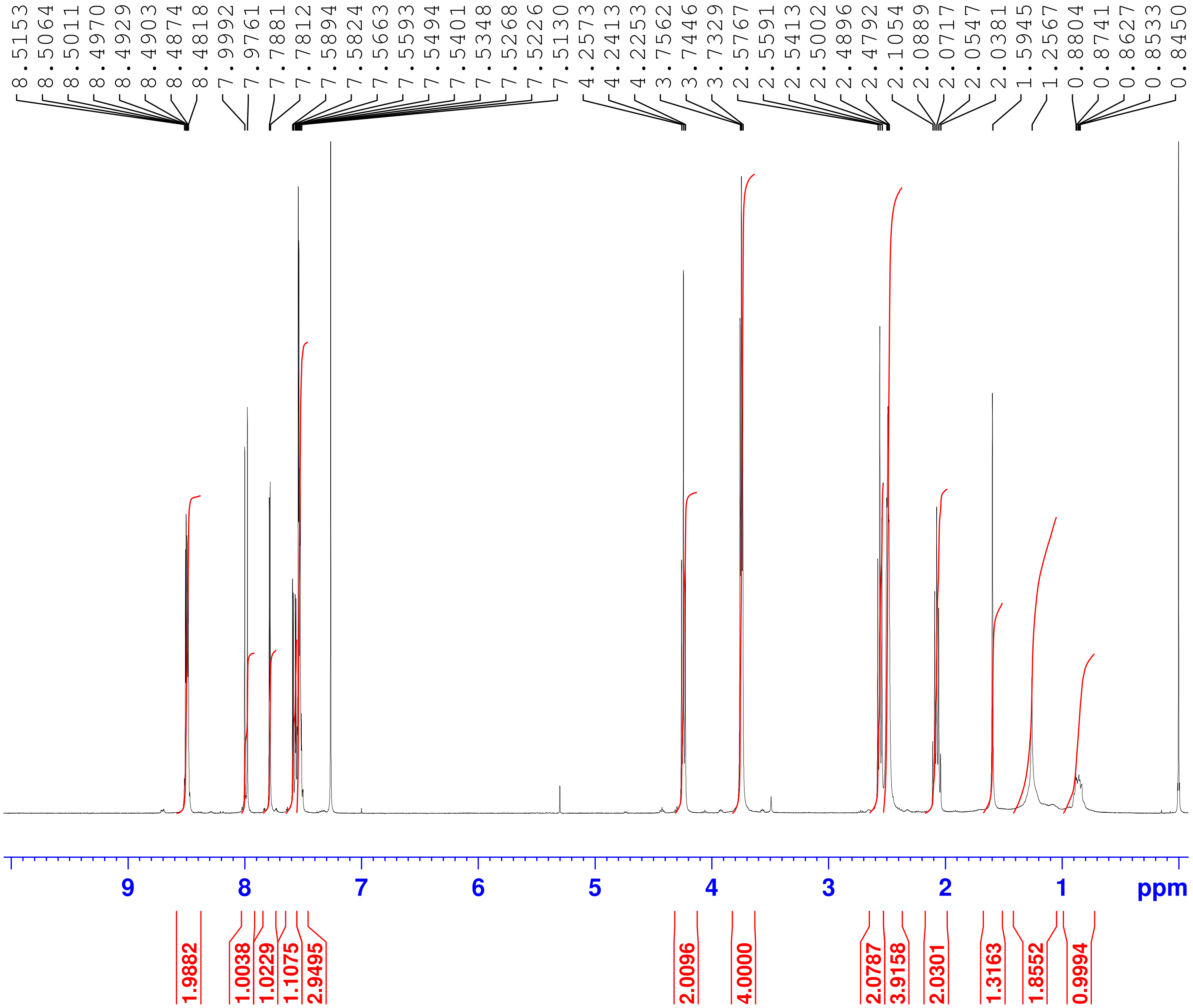

Current Data Parameters
NAME Aug04-2017
EXPNO 12
PROCNO 1

F2 - Acquisition Parameters
Date_ 20170805
Time 6.25 h
INSTRUM spect
PROBHD Z108618_0860 (
PULPROG zg30
TD 65536
SOLVENT CDCl3
NS 64
DS 2
SWH 8012.820 Hz
FIDRES 0.244532 Hz
AQ 4.0894465 sec
RG 176.55
DW 62.400 usec
DE 6.50 usec
TE 298.0 K
D1 1.00000000 sec
TD0 1
SFO1 400.1324708 MHz
NUC1 1H
P1 13.60 usec
PLW1 13.19999981 W

F2 - Processing parameters
SI 65536
SF 400.1300084 MHz
WDW EM
SSB 0
LB 0.30 Hz
GB 0
PC 1.00

Compound 9

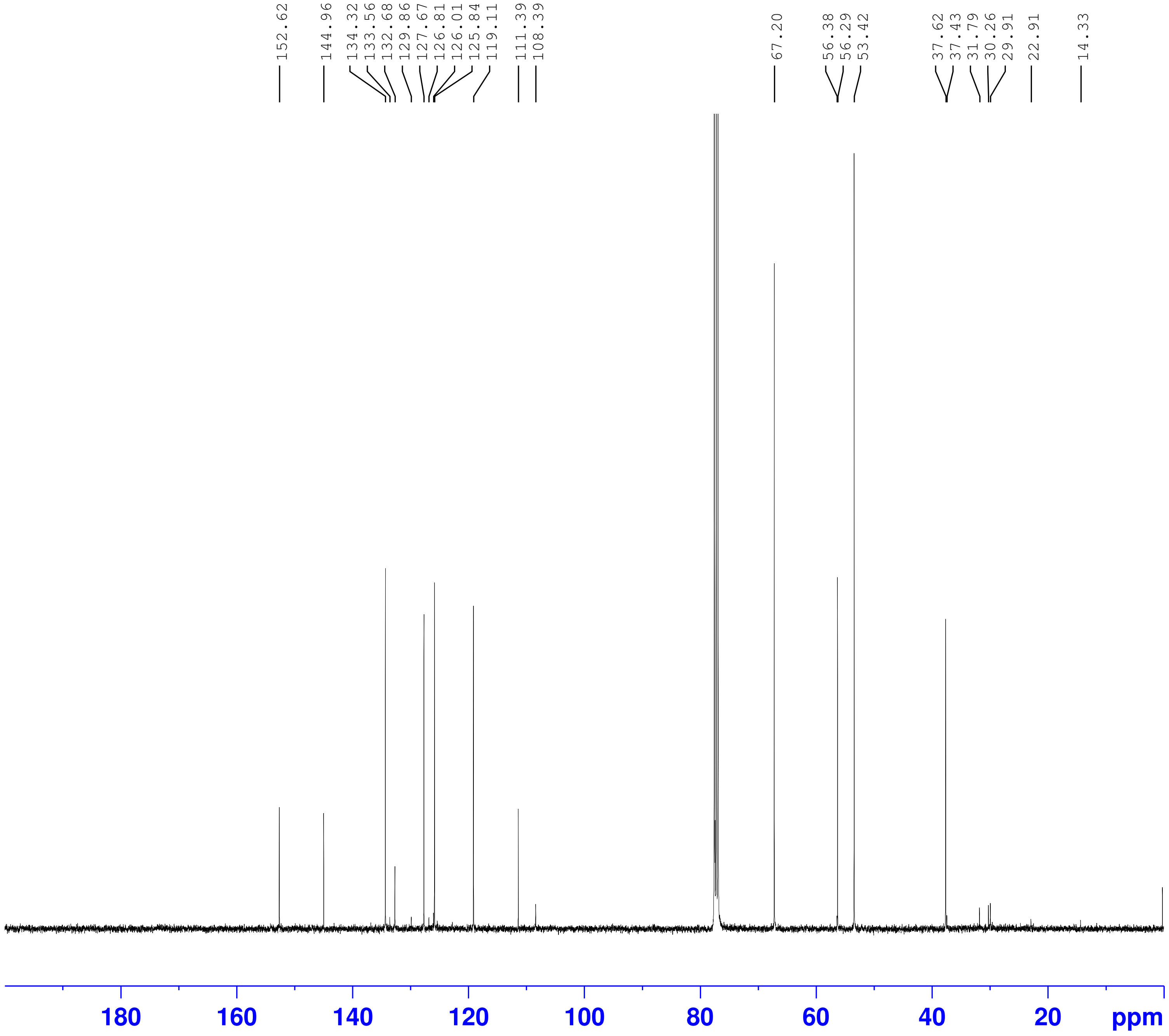

Current Data Parameters
NAME Aug07-2017
EXPNO 10
PROCNO 1

F2 - Acquisition Parameters
Date_ 20170808
Time 4.25 h
INSTRUM spect
PROBHD Z108618_0860 (
PULPROG zgpg50
TD 65536
SOLVENT CDCl3
NS 20000
DS 4
SWH 24038.461 Hz
FIDRES 0.733596 Hz
AQ 1.3631488 sec
RG 198.55
DW 20.800 usec
DE 6.50 usec
TE 298.0 K
D1 0.63999999 sec
D11 0.03000000 sec
TD0 1
SFO1 100.6228298 MHz
NUC1 13C
P1 10.00 usec
PLW1 48.17399979 W
SFO2 400.1316005 MHz
NUC2 1H
CPDPRG[2 waltz16
PCPD2 90.00 usec
PLW2 13.19999981 W
PLW12 0.30142000 W
PLW13 0.15161000 W

F2 - Processing parameters
SI 32768
SF 100.6127479 MHz
WDW EM
SSB 0
LB 1.00 Hz
GB 0
PC 1.40

Compound 3

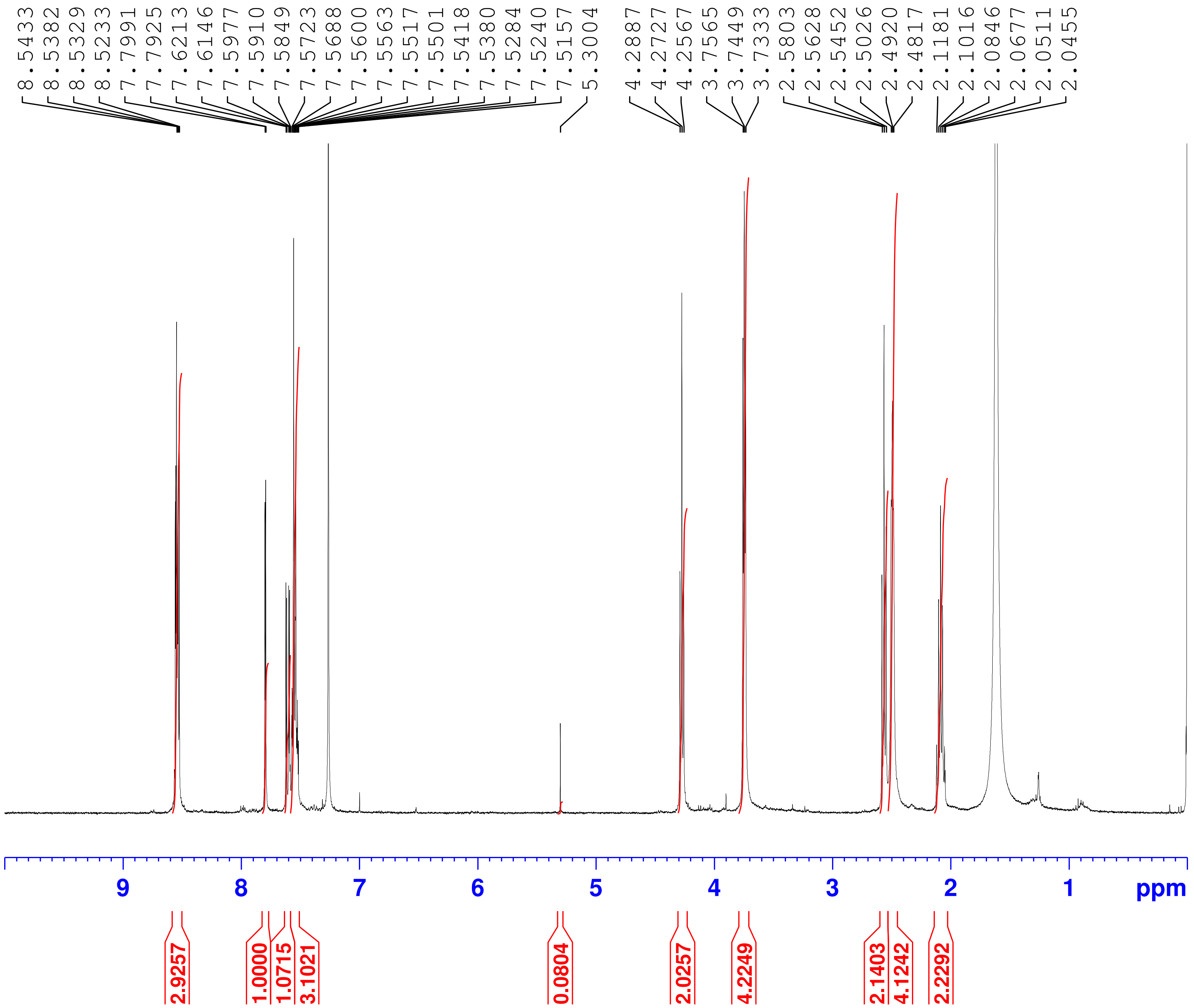

Current Data Parameters
NAME Jan25-2018
EXPNO 8
PROCNO 1

F2 - Acquisition Parameters
Date_ 20180125
Time 14.25 h
INSTRUM spect
PROBHD Z108618_0860 (
PULPROG zg30
TD 65536
SOLVENT CDCl3
NS 64
DS 2
SWH 8012.820 Hz
FIDRES 0.244532 Hz
AQ 4.0894465 sec
RG 198.55
DW 62.400 usec
DE 6.50 usec
TE 298.0 K
D1 1.00000000 sec
TD0 1
SFO1 400.1324708 MHz
NUC1 1H
P1 13.60 usec
PLW1 13.19999981 W

F2 - Processing parameters
SI 65536
SF 400.1300084 MHz
WDW EM
SSB 0
LB 0.30 Hz
GB 0
PC 1.00

Compound 3

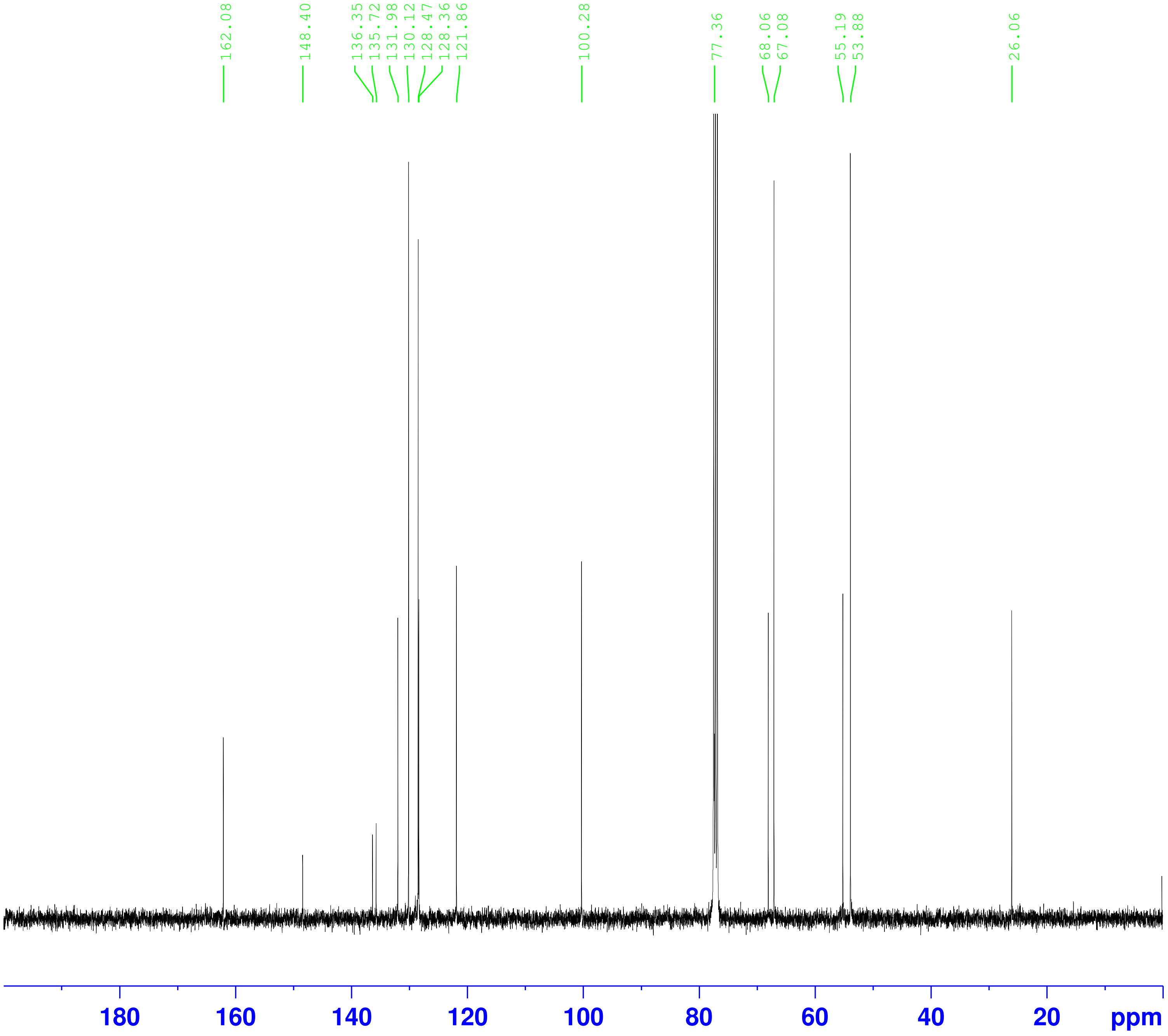


```
Current Data Parameters
NAME           Jan25-2018
EXPNO                  16
PROCNO                  1

F2 - Acquisition Parameters
Date_            20180125
Time                21.07 h
INSTRUM             spect
PROBHD     Z108618_0860 (
PULPROG            zgpg50
TD                  65536
SOLVENT             CDCl3
NS                   8000
DS                      4
SWH             24038.461 Hz
FIDRES           0.733596 Hz
AQ              1.3631488 sec
RG                 198.55
DW                 20.800 usec
DE                   6.50 usec
TE                  298.0 K
D1             0.63999999 sec
D11            0.03000000 sec
TD0                     1
SFO1          100.6228298 MHz
NUC1                  13C
P1                  10.00 usec
PLW1          48.17399979 W
SFO2          400.1316005 MHz
NUC2                   1H
PCPDPRG[2         waltz16
PCPD2               90.00 usec
PLW2          13.19999981 W
PLW12          0.30142000 W
PLW13          0.15161000 W

F2 - Processing parameters
SI                  32768
SF            100.6127551 MHz
WDW                    EM
SSB                     0
LB                   1.00 Hz
GB                      0
PC                   1.40
```
